# Differential associations of serum globulin and albumin-globulin ratio with depression in cancer and non-cancer populations: a cross-sectional study

**DOI:** 10.3389/fpsyt.2025.1523060

**Published:** 2025-04-10

**Authors:** Yichen Zan, Chongxin Guo, Yue Yin, Guanglu Dong

**Affiliations:** Department of Radiation Oncology, The Second Affiliated Hospital of Harbin Medical University, Harbin, China

**Keywords:** depression, cancer, serum globulin, albumin-globulin ratio, biomarkers, logistic regression

## Abstract

**Objective:**

The association of globulin and albumin-globulin ratio (AGR) with depression in cancer and non-cancer populations remains understudied. Therefore, this study aims to investigate this association and potential differences, with a focus on cancer-specific pathophysiology.

**Methods:**

This study utilized data from the National Health and Nutrition Examination Survey (NHANES) conducted from 2005 to 2016. The participants were divided into three tertiles of globulin and AGR to explore more detailed associations. Logistic regression, restricted cubic spline (RCS) curves, and subgroup analyses were conducted to assess the associations. Finally, receiver operating characteristic (ROC) curves were applied to evaluate the predictive performance of globulin and AGR for depression.

**Results:**

After adjusting for covariates, higher globulin levels were significantly associated with an increased incidence of depression in cancer patients (OR=2.53, 95% CI: 1.69-3.80), while a higher AGR was associated with a reduced incidence (OR=0.28, 95% CI: 0.14-0.58). In the non-cancer group, a similar but weaker association was observed: higher globulin levels (OR=1.16, 95% CI: 1.00-1.35) and lower AGR (OR=0.80, 95% CI: 0.62-1.05) were associated with depression. Subgroup analyses suggested that the associations were more stable in cancer populations, while in non-cancer populations, these associations might be influenced by drinking. AUC values indicated that the biomarkers demonstrated good predictive performance.

**Conclusion:**

This study identifies globulin and AGR as novel, cost-effective biomarkers that integrate inflammation and nutrition, providing a convenient and robust means to predict depression, particularly in cancer patients. These findings also offer new perspectives for future dual clinical interventions targeting inflammation and nutrition, as well as experimental research on depression.

## 1 Introduction

Depression, as a major global public health issue, continues to impose an increasing disease burden. According to data from the World Health Organization, depression has evolved from being the third leading cause of the global disease burden in 2008 to a common disorder affecting over 5% of the population (1) and is projected to become the leading cause of disease burden by 2030 (2). Notably, a considerable proportion of depression patients develop treatment-resistant depression (TRD), which not only entails a prolonged disease course and a high risk of relapse but also presents significant challenges in comorbidity management. As highlighted in a recent Italian national expert consensus, the pathological complexity and management heterogeneity of TRD necessitate the establishment of standardized consensus strategies (3). The increasing clinical focus on TRD highlights the necessity of exploring innovative therapeutic strategies and pathophysiological biomarkers, particularly in medically ill populations, such as cancer patients (4).

Advances in medical technology have gradually transformed cancer into a “chronic disease” (5, 6), making the management of depression in cancer survivors an essential clinical concern (7). Studies indicate that 25% of cancer patients experience depression at some stage of the disease (8), and only 27% of those diagnosed with major depression receive standard antidepressant treatment (9). The challenges in identifying and treating depression are significantly heightened in the context of chronic disease comorbidity, underscoring the urgent need for interdisciplinary intervention models and further exploration of underlying pathophysiological mechanisms.

There is a complex interrelationship between cancer and depression. Cancer patients frequently encounter emotional stress and treatment side effects that may trigger or worsen depressive symptoms (10). Biologically, cancer-induced inflammatory responses, endocrine disorders, and neurotransmitter system imbalances are closely related to the onset of depression (11). For example, pancreatic cancer promotes depression through cytokines (12), while paraneoplastic syndromes caused by lung and other cancers disrupt the endocrine and neurotransmitter systems, leading to depressive symptoms (13). Furthermore, individual temperament profiles may play a role in the manifestation and persistence of depressive symptoms in cancer patients, potentially influencing susceptibility to inflammation and stress-related processes (14). Some studies have also shown that depression is related to the occurrence and progression of certain cancers. For instance, a large retrospective cohort study found that depression increased the overall risk of cancer diagnosis by 18%, with particularly significant risks for lung, gastrointestinal, breast, and urinary cancers (15). Other studies have indicated that depression promotes cancer progression and affects prognosis through pathophysiological pathways such as inflammatory factors, immune responses, the HPA axis, and stress responses (16, 17). Additionally, depression may negatively impact the physical and mental health, quality of life, and treatment adherence of cancer patients, leading to poor treatment outcomes and prognosis, and increasing the burden on healthcare systems (18). Therefore, researching depression in cancer patients is essential.

Multiple studies have pointed out that depression is associated with inflammation (19) and the acute phase response (APR) (20). APR is a systemic response of the body to disturbances in internal homeostasis, such as infection, inflammation, immune dysregulation, tissue damage, and tumor growth (21). It involves changes in the immune system, metabolism, behavior, and neuroendocrine function, accompanied by alterations in plasma proteins (such as albumin and globulin) and cytokine levels (22, 23). Although previous studies have examined the relationship between some inflammatory biomarkers (24–26) (such as cytokines, C-reactive protein) and depression, these biomarkers are relatively transient and susceptible to short-term influences, and there are conflicts between different research conclusions. In contrast, globulin and the albumin-globulin ratio (AGR) reflect long-term immune activation, chronic inflammation burden and nutritional metabolism levels, providing unique insights into the relationship between prolonged systemic dysregulation and chronic mental disorders.

Previous research on the relationship between serum albumin and depression has mainly focused on epidemiological studies in the general population (27) and specific clinical groups, such as patients with systemic lupus erythematosus (SLE) (28), elderly stroke patients (29) and patients with chronic liver disease (30), while overlooking cancer as a distinct clinical subgroup. And studies of the association between globulin and depression are limited and based on small sample sizes (31, 32), with no evidence from large populations. In addition, studies of the association between AGR, which reflects plasma protein balance, and depression remain scarce. In particular, no study has systematically compared how serum globulin levels and AGR are associated with depression in cancer and non-cancer populations. And it is worth noting that there is currently no systematic study comparing the association and potential differences between serum globulin levels, albumin-globulin ratios and depression between cancer and non-cancer populations.

This study aims to fill this gap by analyzing globulin levels and AGR in cancer and non-cancer populations based on nationwide samples to explore their potential association with depression. This may help identify high-risk populations for depression, facilitate timely interventions, improve treatment adherence and quality of life in cancer patients, and alleviate the burden on families and the healthcare system. Meanwhile, the differences observed among various populations may reveal underlying pathophysiological mechanisms in the development of depression, providing new directions and clinical insights for further research.

## 2 Methods

### 2.1 Data source

The National Health and Nutrition Examination Survey (NHANES) (https://www.cdc.gov/nchs/nhanes/), conducted by the National Center for Health Statistics (NCHS), is a nationwide assessment initiated in the early 1960s and periodically conducted to assess the health and nutritional status of the U.S. population. This survey employs a complex, stratified sampling design to comprehensively collect demographic data, physical examination results, laboratory test data, health questionnaire responses, and prescription drug usage information. Detailed data content, usage methods, and sampling information are available on the official website. The NHANES study protocol is approved by an institutional review board (IRB), and all participants sign informed consent before the survey. As the data are not directly linked to the participants, they can be used for analysis without additional ethical review, in accordance with NIH policy.

### 2.2 Study populations

To ensure consistency in data measurement methods, we utilized data from six survey cycles spanning 2005-2006 to 2015-2016, encompassing a total of 60,936 participants. Participants completed an in-home interview followed by clinical assessments at a mobile examination center. Participants under 20 years old, lacking globulin and albumin data, without self-reported cancer information, missing depression questionnaire data, or lacking covariate information were excluded from the study. Ultimately, 2,371 cancer patients and 23,094 non-cancer individuals were included in the analysis.

### 2.3 Measurement of globulin, albumin, and the definition of AGR

The data on globulin and AGR were derived from detailed biochemical analyses conducted at the mobile examination center. Globulin is a diverse group of blood proteins involved in substance transport and various immune defense mechanisms, and it is measured by subtracting albumin from total protein. AGR is calculated by dividing the albumin level by the globulin level. These clinical biochemical markers are commonly used to assess liver function, immune status, and nutritional condition. Detailed measurement methods and related technical information can be accessed on the NHANES official website.

### 2.4 Identification of cancer and non-cancer populations

The identification of cancer patients was based on self-reported survey questionnaires. Participants who answered “Yes” to the question “Have you ever been told by a doctor or other health professional that you had cancer or a malignancy of any kind?” were classified as cancer patients, while those who answered “No” were classified as non-cancer individuals.

### 2.5 Assessment of depressive conditions

We assessed the depressive status of participants using the validated PHQ-9 questionnaire. The PHQ-9 is a concise yet effective self-assessment tool for depression, consisting of nine questions scored from 0 to 3, yielding a total score between 0 and 27. Higher scores indicate more severe depressive symptoms. Following prior research on the PHQ-9’s diagnostic accuracy, we defined participants scoring 10 or higher as having depression (33, 34).

### 2.6 Identification of covariates

In line with previous studies, we incorporated several common covariates into this study. The evaluated covariates included gender, age, race, educational level, marital status, poverty income ratio (PIR), body mass index (BMI), smoking status, drinking status, cardiovascular disease (CVD), diabetes, and sleep trouble.

In this study, age was grouped into “20-39 years”, “40-59 years”, and “≥ 60 years”. Race was classified using the original categories. Educational level was categorized as “Less than high school”, “High school”, and “More than high school”. Marital status was divided into three categories: “Never married”, “Married/Living with partner” and “Widowed/Divorced/Separated”. PIR was categorized into three levels: “PIR < 1”, “1 ≤ PIR < 3”, and “PIR ≥ 3”. BMI was categorized as “BMI < 25”, “25 ≤ BMI < 30” and “BMI ≥ 30”. Smoking status was divided into “Non-Smoker” and “Smoker”. Drinking status was categorized as “Non-Drinker” and “Drinker”. Cardiovascular disease was defined as the presence of any self-reported condition among “congestive heart failure”, “coronary heart disease”, “angina/angina pectoris”, “heart attack” or “stroke”. Diabetes was defined based on the question “Doctor told you have diabetes”. Sleep trouble was defined based on the question “Ever told doctor had trouble sleeping.”

### 2.7 Statistical analysis

According to the NHANES official analytical guidelines, this study’s analysis accounted for the complex sampling weights and the clustering and stratification of the sample. Categorical variables were described using unweighted frequencies (weighted percentages), and continuous variables were described using medians (interquartile ranges). The differences between groups were assessed using the chi-squared test with Rao & Scott’s second-order correction and the Wilcoxon rank-sum test for complex survey samples.

A weighted generalized linear model was applied to examine the association between globulin, AGR and depression in cancer and non-cancer populations. To further investigate potential dose-response relationships, we included tertile group variables of transformed globulin and AGR in the analysis. To account for potential confounders and improve model interpretability, we constructed three models with different levels of covariate adjustment: Model 1 was unadjusted, Model 2 adjusted for “gender, age, race, educational level, marital status, and PIR” and Model 3 further adjusted for “BMI, smoking status, drinking status, CVD, diabetes, and sleep trouble”. Restricted cubic spline (RCS) curves and the Wald test were applied, adjusted for different covariates, to explore potential nonlinear associations. Subgroup analyses and interaction tests were further performed with various covariate adjustments to evaluate interaction effects and their robustness. Finally, we evaluated the predictive ability of the globulin and AGR models for depression in cancer and non-cancer populations using receiver operating characteristic (ROC) curves and area under curve (AUC) values.

All analyses were performed using R software version 4.3.1, with a p < 0.05 considered statistically significant.

## 3 Results

### 3.1 Baseline characteristics

Baseline demographics revealed significant differences between depressed and non-depressed individuals in both cancer and non-cancer groups (**Table 1**). In the cancer group, depressed patients were more likely to be female, aged 40-59 years, have lower PIR, smoke, and have sleep troubles. Additionally, higher globulin levels and lower AGR were observed in depressed cancer patients. Similar trends were observed in the non-cancer group, with significant differences in gender, age, poverty status, smoking, and sleep trouble, although the differences in globulin and AGR were less pronounced compared to the cancer group.

**Table 1:** Basic characteristics of participants.

	Cancer group N = 2371			Non-Cancer group N = 23094		
Characteristic	Non-Depression N = 2125 (91%)^1^	Depression N = 246 (8.8%)^1^	p-value^2^	Non-Depression N = 21116 (93%)^1^	Depression N = 1978 (7.4%)^1^	p-value^2^
**Gender**			**0.009**			**<0.001**
Male	1,037 (44%)	78 (32%)		10,685 (51%)	722 (36%)	
Female	1,088 (56%)	168 (68%)		10,431 (49%)	1,256 (64%)	
**Age group**			**<0.001**			**0.003**
20-39 years	130 (7.2%)	37 (16%)		7,940 (40%)	678 (37%)	
40-59 years	429 (29%)	90 (47%)		7,063 (39%)	807 (44%)	
≥ 60 years	1,566 (63%)	119 (37%)		6,113 (21%)	493 (19%)	
**Race group**			**<0.001**			**<0.001**
Mexican American	111 (1.8%)	32 (4.7%)		3,557 (8.7%)	304 (8.1%)	
Non-Hispanic Black	280 (4.5%)	32 (6.6%)		4,369 (11%)	451 (14%)	
Non-Hispanic White	1,544 (89%)	151 (79%)		9,159 (69%)	833 (64%)	
Other Hispanic	119 (2.1%)	14 (2.1%)		1,928 (5.0%)	265 (7.9%)	
Other/multiracial	71 (2.8%)	17 (7.6%)		2,103 (6.9%)	125 (6.0%)	
**Education group**			**0.001**			**<0.001**
Less than high school	402 (11%)	84 (22%)		4,962 (15%)	705 (26%)	
High School	473 (20%)	54 (25%)		4,807 (22%)	484 (27%)	
More than high school	1,250 (69%)	108 (53%)		11,347 (62%)	789 (47%)	
**PIR group**			**<0.001**			**<0.001**
PIR < 1	258 (7.4%)	90 (29%)		4,194 (13%)	776 (30%)	
1 ≤ PIR < 3	889 (33%)	114 (45%)		8,719 (35%)	880 (45%)	
PIR ≥ 3	978 (60%)	42 (26%)		8,203 (52%)	322 (25%)	
**Marital group**			**<0.001**			**<0.001**
Never Married	119 (5.7%)	24 (7.5%)		3,917 (18%)	428 (22%)	
Married/living with partner	1,334 (67%)	106 (52%)		13,038 (66%)	918 (49%)	
Widowed/ divorced/separated	672 (27%)	116 (41%)		4,161 (16%)	632 (30%)	
**BMI group**			**0.049**			**<0.001**
BMI < 25	592 (30%)	62 (24%)		6,185 (30%)	481 (26%)	
25 ≤ BMI < 30	762 (35%)	70 (30%)		7,155 (34%)	516 (26%)	
BMI ≥ 30	771 (35%)	114 (46%)		7,776 (36%)	981 (48%)	
**Drink group**			0.700			0.300
Non-Drinker	607 (24%)	73 (22%)		5,920 (22%)	545 (23%)	
Drinker	1,518 (76%)	173 (78%)		15,196 (78%)	1,433 (77%)	
**Smoke group**			**<0.001**			**<0.001**
Non-Smoker	977 (47%)	77 (29%)		11,952 (56%)	803 (39%)	
Smoker	1,148 (53%)	169 (71%)		9,164 (44%)	1,175 (61%)	
**CVD group**			**0.002**			**<0.001**
Non-CVD	1,647 (83%)	167 (72%)		19,345 (93%)	1,636 (86%)	
CVD	478 (17%)	79 (28%)		1,771 (6.6%)	342 (14%)	
**Diabetes group**			0.400			**<0.001**
Non-Diabetes	1,672 (82%)	175 (79%)		18,398 (90%)	1,556 (82%)	
Diabetes	453 (18%)	71 (21%)		2,718 (9.7%)	422 (18%)	
**Sleep group**			**<0.001**			**<0.001**
Non-Sleep trouble	1,431 (65%)	83 (28%)		16,634 (77%)	864 (39%)	
Sleep trouble	694 (35%)	163 (72%)		4,482 (23%)	1,114 (61%)	
**ALB**	4.20 (4.00, 4.40)	4.20 (4.00, 4.43)	0.200	4.30 (4.10, 4.50)	4.20 (4.00, 4.40)	**<0.001**
**GLB**	2.70 (2.40, 2.90)	2.90 (2.60, 3.10)	**<0.001**	2.80 (2.50, 3.10)	2.80 (2.60, 3.20)	**<0.001**
**AGR**	1.60 (1.41, 1.78)	1.47 (1.26, 1.68)	**<0.001**	1.56 (1.38, 1.75)	1.48 (1.29, 1.67)	**<0.001**
**GLB Tertile group**			**<0.001**			**<0.001**
Tertile 1	678 (39%)	51 (24%)		6,204 (37%)	496 (30%)	
Tertile 2	745 (37%)	72 (30%)		7,851 (37%)	708 (37%)	
Tertile 3	702 (24%)	123 (47%)		7,061 (25%)	774 (33%)	
**AGR Tertile group**			**<0.001**			**<0.001**
Tertile 1	873 (32%)	136 (49%)		8,959 (33%)	1,013 (44%)	
Tertile 2	695 (34%)	68 (31%)		6,718 (33%)	573 (31%)	
Tertile 3	557 (34%)	42 (21%)		5,439 (34%)	392 (25%)	

^1^N (unweighted) (%); Median (IQR).

^2^chi-squared test with Rao & Scott’s second-order correction; Wilcoxon rank-sum test for complex survey samples.

ALB, albumin; GLB, globulin; AGR: albumin-globulin ratio. P-values less than 0.05 are shown in bold.

### 3.2 Globulin is positively associated with depression in cancer and non-cancer groups

The regression analysis results in **Table 2** indicate that elevated globulin levels are associated with an increased risk of depression in both cancer and non-cancer populations, although the effect sizes differ. In the cancer group, the odds ratios (ORs) across all models ranged from 2.33 to 2.61 (p<0.001). After full adjustment for covariates (Model 3), each unit increase in globulin levels was associated with approximately a 2.5-fold higher risk of depression in cancer patients (OR=2.53, 95% CI: 1.69–3.80, p<0.001). Further analysis showed that in the fully adjusted model, individuals in the highest globulin tertile (Tertile 3) had a 3.06-fold higher risk of depression compared to those in the lowest tertile (Tertile 1) (OR=3.06, 95% CI: 1.78–5.26, p<0.001), with a significant trend test (P trend<0.001). This suggests a relatively clear dose-response relationship between globulin levels and depression risk in cancer patients. In the non-cancer group, although a similar positive association was observed, the ORs were lower (1.16 to 1.55; p-values ranging from <0.001 to 0.050). Furthermore, in the non-cancer group, the risk increase for individuals in the highest globulin tertile (Tertile 3) was less pronounced compared to the cancer group (OR: 1.18 to 1.60, p-values ranging from <0.001 to 0.038). After adjusting for all covariates (Model 3), individuals in the highest globulin tertile (Tertile 3) had a 1.18-fold higher risk of depression compared to those in the lowest tertile (Tertile 1) (OR=1.18, 95% CI: 1.01–1.39, p=0.038), and the trend test was no longer significant (P trend=0.070), suggesting a more complex association.

**Table 2:** Associations of globulin and AGR with depression in cancer and non-cancer populations.

Variable group	Group	Variable	Model 1		Model 2		Model 3	
			OR (95% CI)	p-value	OR (95% CI)	p-value	OR (95% CI)	p-value
**GLB**	**Cancer group**	**GLB**	2.61 (1.95, 3.49)	**<0.001**	2.33 (1.65, 3.29)	**<0.001**	2.53 (1.69, 3.80)	**<0.001**
		GLB tertile group						
		**Tertile** 1	Ref		Ref		Ref	
		**Tertile** 2	1.34 (0.85, 2.12)	0.200	1.28 (0.77, 2.13)	0.300	1.32 (0.78, 2.24)	0.300
		**Tertile** 3	3.27 (2.03, 5.28)	**<0.001**	2.91 (1.70, 4.97)	**<0.001**	3.06 (1.78, 5.26)	**<0.001**
		**p for trend**	**<0.001**		**<0.001**		**<0.001**	
	**Non-Cancer group**	**GLB**	1.55 (1.36, 1.76)	**<0.001**	1.17 (1.02, 1.36)	**0.030**	1.16 (1.00, 1.35)	0.050
		GLB tertile group						
		**Tertile** 1	Ref		Ref		Ref	
		**Tertile** 2	1.22 (1.03, 1.45)	**0.022**	1.05 (0.88, 1.25)	0.600	1.06 (0.88, 1.27)	0.500
		**Tertile** 3	1.60 (1.37, 1.87)	**<0.001**	1.19 (1.01, 1.40)	**0.033**	1.18 (1.01, 1.39)	**0.038**
		**p for trend**	**<0.001**		0.059		0.070	
**AGR**	**Cancer group**	**AGR**	0.24 (0.14, 0.42)	**<0.001**	0.31 (0.17, 0.57)	**<0.001**	0.28 (0.14, 0.58)	**<0.001**
		AGR tertile						
		**Tertile** 1	Ref		Ref		Ref	
		**Tertile** 2	0.59 (0.39, 0.89)	**0.012**	0.70 (0.43, 1.14)	0.150	0.69 (0.42, 1.14)	0.150
		**Tertile** 3	0.40 (0.24, 0.66)	**<0.001**	0.46 (0.26, 0.81)	**0.008**	0.44 (0.24, 0.81)	**0.008**
		**p for trend**	**<0.001**		**0.020**		**0.024**	
	**Non-Cancer group**	**AGR**	0.42 (0.32, 0.54)	**<0.001**	0.70 (0.54, 0.91)	**0.009**	0.80 (0.62, 1.05)	0.110
		AGR tertile						
		**Tertile** 1	Ref		Ref		Ref	
		**Tertile** 2	0.72 (0.62, 0.83)	**<0.001**	0.87 (0.75, 1.02)	0.086	0.93 (0.79, 1.10)	0.400
		**Tertile** 3	0.56 (0.48, 0.66)	**<0.001**	0.80 (0.67, 0.96)	**0.018**	0.89 (0.74, 1.07)	0.200
		**p for trend**	**<0.001**		**0.044**		0.400	

Model 1: Unadjusted.

Model 2: Adjusted for gender, age, race, educational level, marital status, and PIR.

Model 3: Adjusted as for model 2, additionally adjusted BMI, smoking status, drinking status, CVD, diabetes, and sleep trouble.

OR, Odds Ratio; CI, Confidence Interval; Ref, reference; GLB, globulin; AGR, albumin-globulin ratio. P-values less than 0.05 are shown in bold.

### 3.3 AGR is negatively associated with depression in cancer and non-cancer groups

As shown in **Table 2**, for the cancer group, a higher AGR was negatively associated with depression, with ORs ranging from 0.24 to 0.31 (p<0.001). After adjusting for all variables (Model 3), each unit increase in AGR was associated with a 72% lower risk of depression (OR=0.28, 95% CI: 0.14–0.58, p<0.001). Compared to the reference tertile (Tertile 1), individuals in the highest AGR tertile (Tertile 3) had a significantly 56% lower risk of depression (Model 3, OR=0.44, 95% CI: 0.24–0.81, p=0.008), with a significant trend observed (P trend=0.024). However, for the non-cancer group, the protective effect of the AGR was obviously reduced (OR: 0.42 to 0.80, p ranging from <0.001 to 0.110). This weakening of the association was also reflected in the comparisons between tertiles and the trend test.

### 3.4 Restricted cubic spline curves

**Figure 1:**
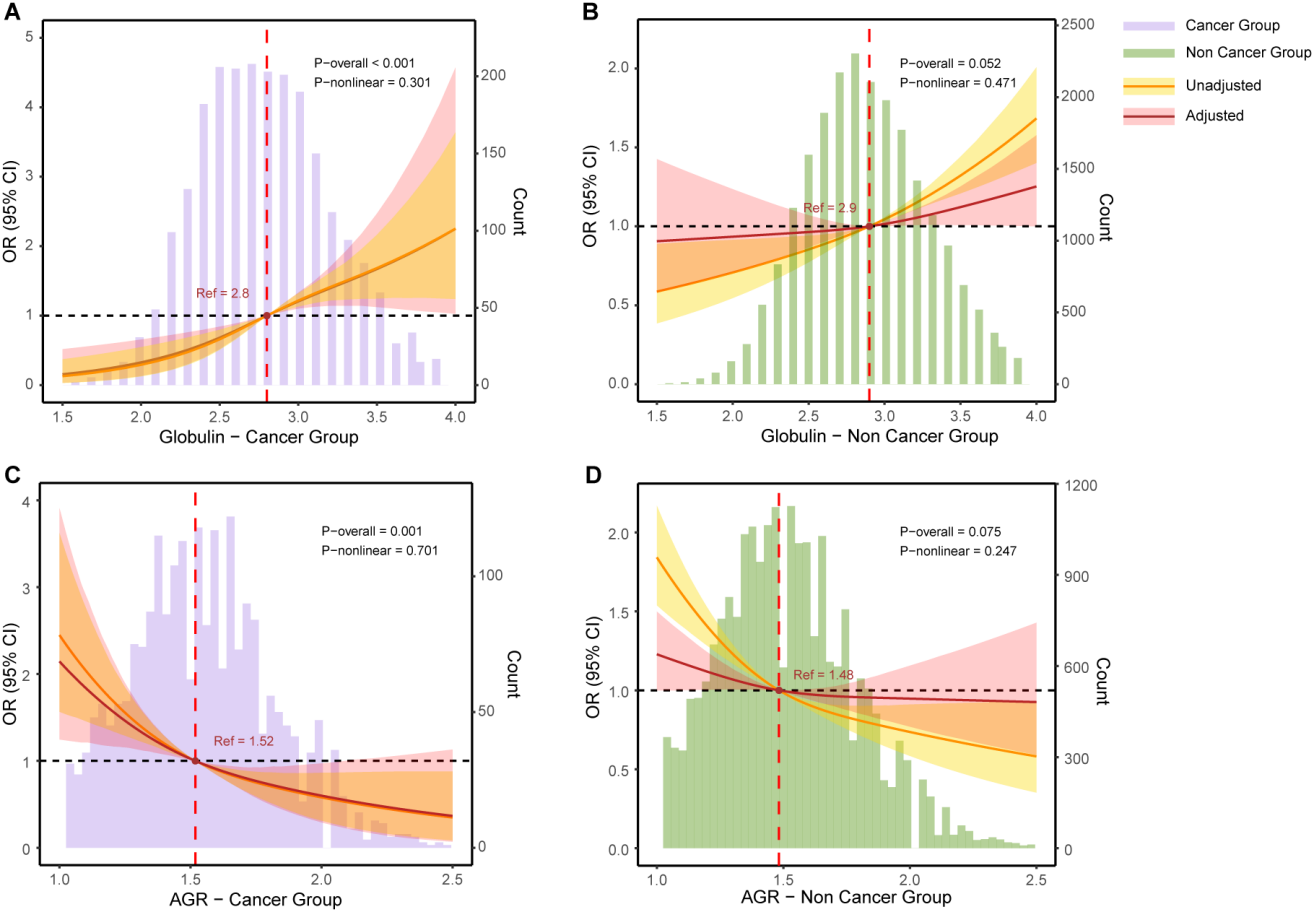
The restricted cubic splines for the associations of globulin and AGR with depression. **(A)** Globulin was significantly and linearly positively associated with depression in the cancer group (P overall < 0.001, P nonlinear = 0.301). **(B)** After adjusting for all covariates, globulin showed a borderline significant positive association with depression in the non-cancer group (P overall = 0.052, P nonlinear = 0.471). **(C)** In the cancer group, AGR was significantly and linearly negatively associated with depression (P overall = 0.001, P nonlinear = 0.701). **(D)** After adjusting for all covariates, AGR showed a borderline significant negative association with depression in the non-cancer group (P overall = 0.075, P nonlinear = 0.247). The purple bar graphs represent the distribution of globulin and AGR in the cancer group, while the green bar graphs represent their distribution in the non-cancer group. The restricted cubic spline (RCS) curves represent the relative odds ratio (OR) as solid lines, with the shaded area indicating the 95% confidence interval (CI). The yellow curve represents the unadjusted model, while the red curve represents adjustments for the covariates listed in Model 3 of **Table 2**.

To further investigate the potential nonlinear relationships between globulin and AGR with depression in cancer and non-cancer populations, we employed RCS curves for analysis and visualization. As shown in **Figure 1A**, the positive association between globulin levels and depression in cancer patients remained consistent before and after adjustment for covariates (P-nonlinear = 0.301). In contrast, in the non-cancer population (**Figure 1B**), the RCS plot showed a nonlinear trend after adjustment for covariates (red curve), although the p-value was not significant (the risk of depression increased slowly until globulin levels reached approximately 2.9, after which it increased rapidly; P-nonlinear = 0.471). A similar pattern was observed for AGR. As shown in **Figure 1C**, the association of AGR remained stable before and after covariate adjustment in cancer patients (P-nonlinear = 0.701). In non-cancer individuals (**Figure 1D**), the RCS plot for AGR also exhibited a nonlinear trend after adjustment for covariates (red curve), although the p-value was not significant (OR decreased rapidly before AGR reached 1.48 and then remained relatively stable; P-nonlinear = 0.247). These findings highlight the different patterns of association between globulin levels and AGR with depression in cancer and non-cancer populations.

### 3.5 Subgroup analyses

**Figure 2:**
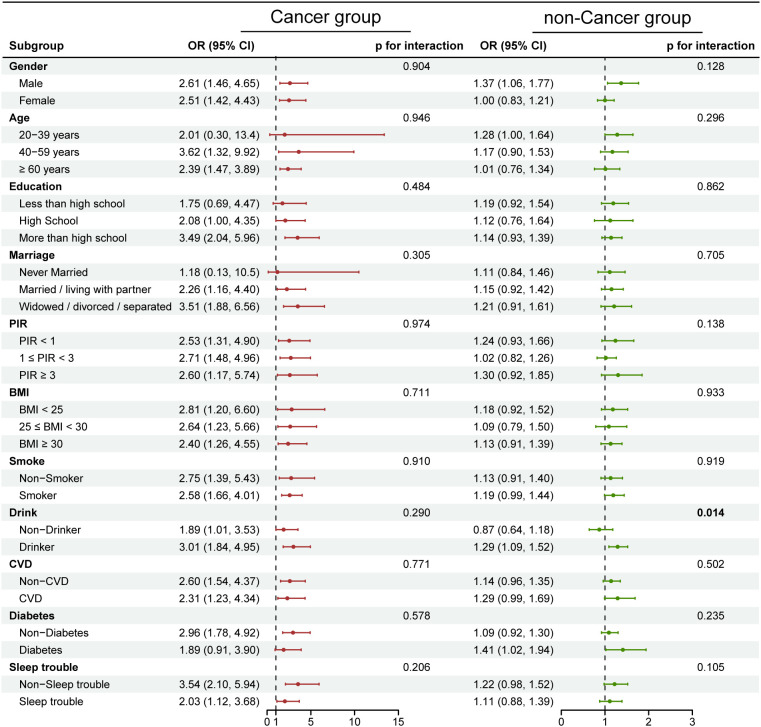
Subgroup analysis of the relationship between globulin and depression in cancer and non-cancer populations. Adjustments were made for the covariates in Model 3 (sex, age, race, educational level, marital status, PIR, BMI, smoking, drinking, CVD, diabetes, sleep trouble), and stratification variables themselves were not adjusted within the respective strata. The red dots represent OR values for the cancer group, and the green dots represent OR values for the non-cancer group, with the lines indicating 95% CI. In the cancer population, no significant interaction was observed between globulin and different subgroups. However, in the non-cancer population, interaction was observed in the drinking subgroup (p for interaction = 0.014). P-values less than 0.05 are shown in bold.

**Figure 3:**
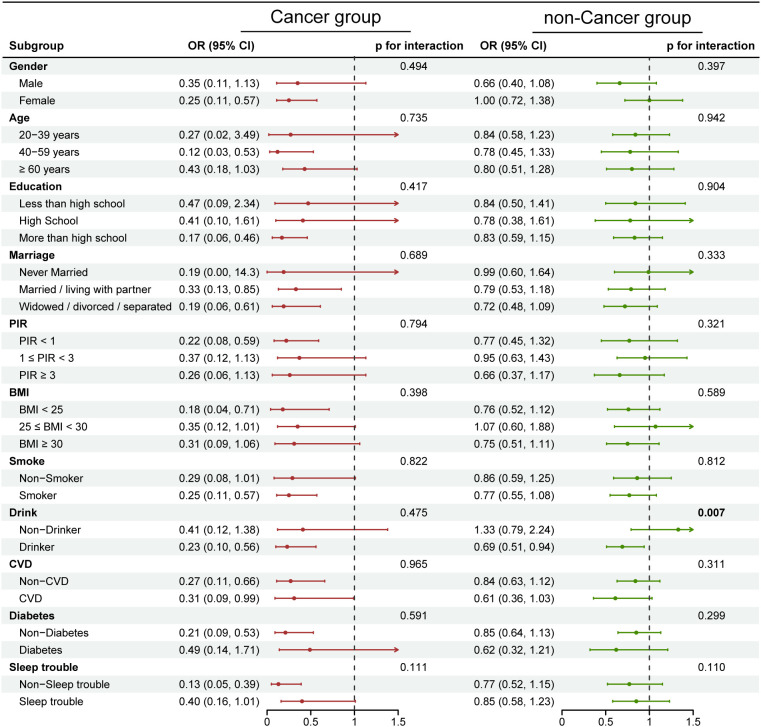
Subgroup analysis of the relationship between AGR and depression in cancer and non-cancer populations. Each stratification factor is adjusted for the covariates from model 3, except for the stratification factor itself. The red dots represent OR values for the cancer group, and the green dots represent OR values for the non-cancer group, with the lines indicating the 95% CI. In the cancer population, no significant interaction was observed between the different subgroups. In the non-cancer population, interaction was observed in the drinking subgroup (p for interaction = 0.007). P-values less than 0.05 are shown in bold.

We conducted subgroup analyses and interaction tests to explore whether the associations of globulin and AGR with depression in cancer and non-cancer populations differed across various subgroups. The results in **Figure 2** and **Supplementary Table S1** indicate that in each subgroup of the cancer population, the association between globulin and depression remained stable even after adjusting for different covariates. However, an interaction was observed in the drinking subgroup of the non-cancer population (interaction p-values ranged from 0.009 to 0.023 with different covariate adjustments). In the drinking group, each unit increase in globulin was associated with a 29% higher risk of depression, whereas this association was reversed in the non-drinking group (drinking group OR=1.29, 95% CI: 1.09–1.52; non-drinking group OR=0.87, 95% CI: 0.64–1.18). Furthermore, AGR (**Figure 3**, **Supplementary Table S2**) exhibit no significant interaction in any subgroup of the cancer population, regardless of covariate adjustment. However, in the non-cancer population, an interaction was observed in the drinking subgroup (interaction p-values ranged from 0.007 to 0.018 with different covariate adjustments). Among drinkers, each unit increase in AGR was associated with a 31% lower risk of depression. Similarly, this association also changed in non-drinkers (drinking group OR=0.69, 95% CI: 0.51–0.94; non-drinking group OR=1.33, 95% CI: 0.79–2.24). Notably, in cancer patients, although the interaction was not statistically significant (Globulin: p for interaction=0.290; AGR: p for interaction=0.475), there was a substantial difference in the effect value between the drinking and non-drinking groups (Globulin: OR=3.01 vs. 1.89; AGR: OR=0.23 vs. 0.41). These results indicate that in the non-cancer population, drinking modifies the association between globulin, AGR, and depression. Conversely, in the cancer population, this association remains highly stable and unaffected by different subgroups.

### 3.6 Receiver operating characteristic curves

**Figure 4:**
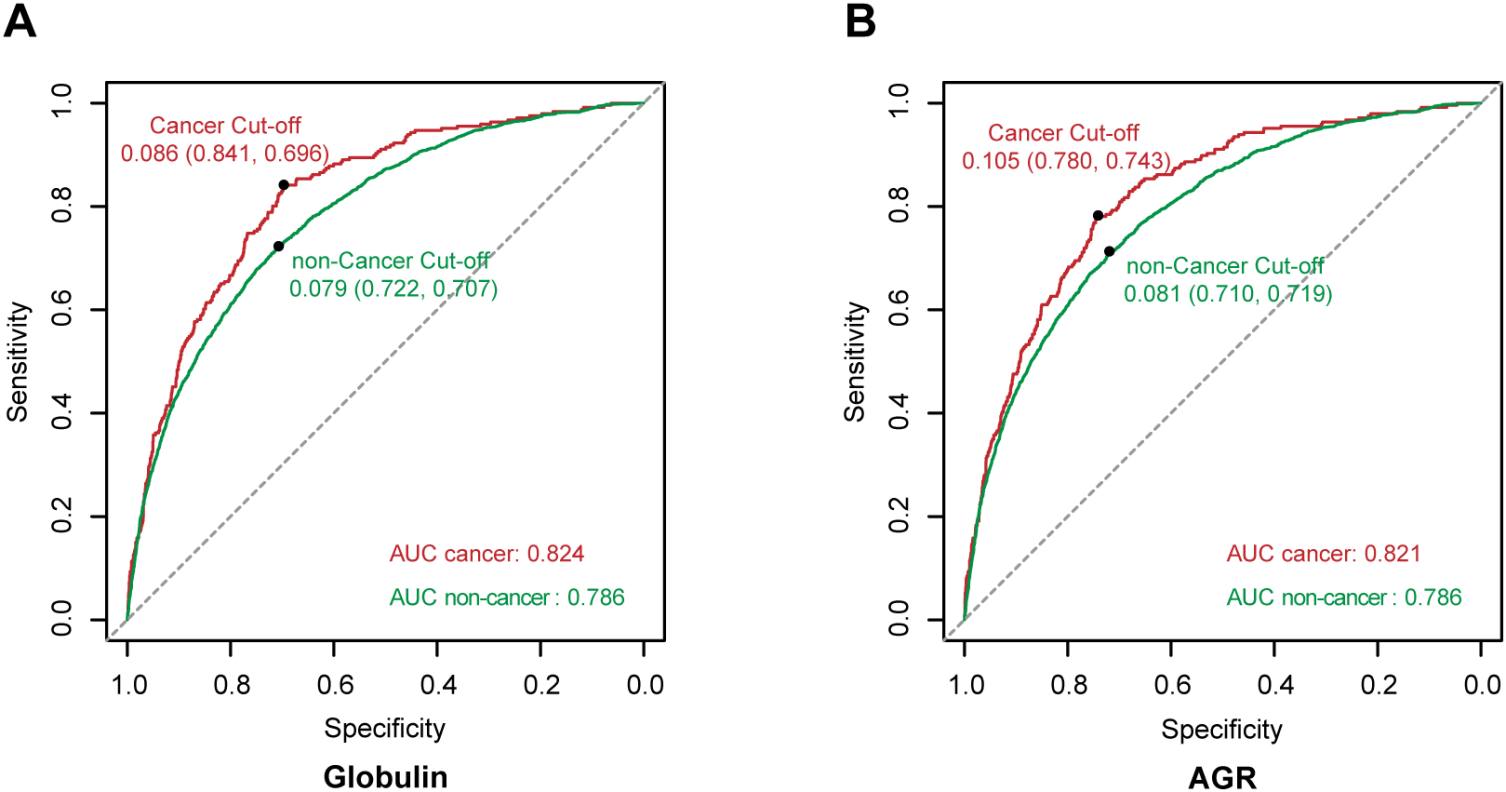
ROC curves for the fully adjusted models of globulin and AGR. **(A)** ROC curves and AUC values of the globulin model. The cut-off for the predictive value in the cancer group is 0.086 (sensitivity: 0.841, specificity: 0.696), while in the non-cancer group it is 0.079 (sensitivity: 0.722, specificity: 0.707). **(B)** ROC curves and AUC values of the AGR model. The cut-off for the predictive value in the cancer group is 0.105 (sensitivity: 0.780, specificity: 0.743), while in the non-cancer group it is 0.081 (sensitivity: 0.710, specificity: 0.719). Red represents the cancer group, and green represents the non-cancer group.

To further assess the predictive ability of the model 3, we utilized receiver operating characteristic (ROC) curves and area under the curve (AUC) values for visualization and evaluation. As shown in **Figure 4**, both globulin and AGR demonstrated good predictive ability for depression in cancer and non-cancer populations (Globulin: AUC cancer = 0.824, AUC non-cancer = 0.786; AGR: AUC cancer = 0.821, AUC non-cancer = 0.786). Although we acknowledge that its predictive performance is slightly lower than that of classic psychological scales such as the PHQ-9 (AUC~0.88) (35) and the BDI-II (AUC~0.85) (36), it can serve as a complementary tool because it is based on objective blood test markers. Furthermore, for asymptomatic individuals or those unwilling to undergo psychological assessment, these biomarkers may serve as effective adjunctive screening tools.

## 4 Discussion

This study aimed to investigate the associations of serum globulin and AGR with depression in cancer and non-cancer populations. Our findings indicate that higher serum globulin levels are significantly positively correlated with depression risk in both cancer and non-cancer groups, with a stronger correlation observed in the cancer population. Conversely, higher AGR is significantly negatively correlated with depression, suggesting a potential protective effect, which is more pronounced in cancer patients compared to the non-cancer group. The results of the RCS analysis underline the complexity of these associations. Regardless of whether covariates are adjusted, the globulin levels and AGR exhibit a stable linear relationship with depression in cancer patients. Whereas in the non-cancer population, although the p-value for the nonlinearity test was not significant, the RCS plot suggested possible nonlinear associations in globulin and AGR, which may require further research in the future. The initial increase in AGR showed a more pronounced risk reduction, but after reaching 1.48, the protective effect gradually weakened. Similarly, the ORs exhibit different magnitudes of change when globulin levels were above and below 2.9.

The heterogeneity observed in the associations of serum globulin and AGR with depression in cancer and non-cancer populations in this study may stem from the cascading effects of cancer-specific pathophysiological networks and distinct mechanisms underlying mental health. The persistent inflammatory state and stress response induced by the tumor microenvironment may serve as key driving mechanisms. For instance, tumor-associated macrophages (TAMs) secrete pro-inflammatory cytokines such as IL-6 and TNF-α, triggering neuroinflammatory responses (37). This effect is significantly attenuated in non-cancer populations due to the absence of persistent stimulation and may rely on distinct compensatory mechanisms, resulting in a possible threshold effect relationship between globulin and depression. In particular, cancer cachexia may exacerbate the pathological effects of this process. For example, hypoalbuminemia not only impairs antioxidant stress capacity (38), but may also reduce the efficiency of tryptophan transport (39) across the blood-brain barrier, limiting the availability of serotonin precursors and increasing the risk of depression. Additionally, the inflammatory response and neurotoxicity induced by anti-tumor drugs, as well as the substantial psychological distress experienced by cancer patients, are also crucial factors contributing to the observed differences in association.

Subgroup analysis revealed heterogeneity in the associations of globulin and AGR with depression across different subgroups in cancer and non-cancer populations. In the non-cancer population, drinking modified the relationship between globulin, AGR, and depression (Globulin: interaction p=0.014; AGR: interaction p=0.007). This may be due to the synergistic effects of oxidative stress, neuroinflammation, neurotransmitter system dysregulation, and hypothalamic-pituitary-adrenal (HPA) axis dysfunction induced by drinking, which collectively enhance the positive association between globulin and depression (drinking group OR=1.29, 95% CI: 1.09–1.52; non-drinking group OR=0.87, 95% CI: 0.64–1.18). Conversely, AGR may exert a stronger protective effect in this adverse microenvironment (drinking group OR=0.69, 95% CI: 0.51–0.94; non-drinking group OR=1.33, 95% CI: 0.79–2.24). Additionally, in cancer patients which exposed to a similarly adverse microenvironment, stronger detrimental/protective effects of globulin and AGR were also observed in the drinking group (Globulin: drinking group OR=3.01, 95% CI: 1.84–4.95; non-drinking group OR=1.89, 95% CI: 1.01–3.53; AGR: drinking group OR=0.23, 95% CI: 0.10–0.56; non-drinking group OR=0.41, 95% CI: 0.12–1.38). However, the impact of cancer itself may have outweighed that of alcohol consumption, resulting in no significant modification of the association (globulin interaction p=0.290, AGR interaction p=0.475). Clinically, in the non-cancer population, particular attention should be paid to globulin and AGR levels in the drinking group. For cancer patients, both drinkers and non-drinkers should be monitored; however, given the larger effect value (Globulin: OR=3.01 vs. 1.89; AGR: OR=0.23 vs. 0.41), more attention should be directed toward the drinking group.

Although CRP and IL-6 are well-established inflammatory markers that have been linked to depression in multiple studies, their specificity for depression is constrained by associations with various physical conditions. In contrast, AGR integrates inflammatory status (via globulin) and nutritional status (via albumin), providing a dual mechanism for assessing depression risk and potentially making it more suitable for predicting depression in specific populations such as cancer patients. As illustrated by the ROC curve, AGR demonstrated superior predictive performance for depression in the cancer population (AUC cancer=0.821, AUC non-cancer=0.786).

Additionally, in clinical practice, inflammatory markers such as CRP and IL-6 are often additional monitoring parameters. Our findings suggest that globulin and AGR can serve as novel biomarkers for depression risk stratification. In oncological clinical practice, routine blood tests already include albumin levels and AGR, enabling clinicians to identify high-risk patients with minimal additional cost, prioritize psychological health screening, and monitor treatment adherence. In the general non-cancer population, these biomarkers (e.g., globulin > 2.9 g/dL and AGR < 1.48) may serve as cost-effective adjuncts to existing depression screening tools (e.g., PHQ-9), aiding in diagnosis and longitudinal monitoring. Future multicenter prospective studies can explore optimal predictive thresholds for different subgroups and validate their sensitivity and specificity across independent cohorts. In addition, interventional studies could investigate whether combining inflammation modulation with nutritional interventions reduces depression incidence and improves treatment outcomes in high-risk populations.

From the pathophysiological mechanism of depression, complement proteins are essential components of globulins, and their activation under chronic stress can promote microglial activation (40). Central immune pathways such as excessive microglial activation and astrocytic dysfunction, along with neuroendocrine dysregulation induced by hyperactivation of the HPA axis and sympathetic nervous system, can contribute to the onset and progression of depression (41–44). Additionally, the imbalance of the kynurenine metabolic pathway induced by prolonged stress, leading to increased quinolinic acid (44), is closely associated with depression. As the primary carrier of tryptophan (45), albumin is related to the availability of tryptophan (46) and may affect the transition of tryptophan metabolism to the kynurenine pathway, thereby reducing the production of the neurotoxic metabolite quinolinic acid. Moreover, its free radical scavenging and antioxidant properties (47, 48) can suppress lipid peroxidation and reactive oxygen species production induced by quinolinic acid, thereby antagonizing neurotoxicity. The bidirectional communication between central and peripheral immune-related cells and cytokines further promotes depression, while Th17 cells compromise the structure of the blood-brain barrier (BBB) (49), exacerbating this pathogenic interaction. Pathologically elevated globulins may contain autoantibodies targeting brain antigens (50) (e.g., NMDAR-Ab), which can penetrate the compromised BBB, triggering neuroinflammation and neuronal damage. Complement proteins within globulins act synergistically with autoantibodies, activating microglia and damaging astrocytes upon entry into the central nervous system, ultimately initiating a cascade of neuroinflammatory responses that disrupt synaptic plasticity and exacerbate depression. AGR, as an indicator of the balance between albumin and globulins, plays a pivotal integrative role in these processes.

Our findings align with previous studies that have identified a connection between certain serum proteins and depression. For instance, studies have demonstrated a negative association between albumin and depression in stroke survivors, patients with chronic liver disease, and adolescents with systemic lupus erythematosus, hypothesizing that this association may be linked to oxidative stress and inflammatory responses (27–30). Similar to our findings, studies by Michael Maes and Cai Song et al. have shown that α-globulin levels are higher in patients with depression (31, 32). However, our study extends these findings by specifically examining cancer and non-cancer populations, revealing a stronger association between globulin levels and depressive symptoms in cancer patients. This distinction is crucial, as it highlights the unique physiological and psychological stress that cancer patients face, which may amplify the impact of inflammation on mental health (51). Additionally, our study analyzed AGR, which has been overlooked by other studies, offering new insights into how the balance between albumin and globulin affects depressive symptoms. Therefore, this study builds on previous research by further demonstrating that nutritional and inflammatory markers, including globulin and the AGR, play a critical role in the pathophysiology of depression, particularly among vulnerable populations such as cancer patients.

We utilized a large and diverse sample from the National Health and Nutrition Examination Survey (NHANES), enhancing the generalizability of our findings. The use of RCS enabled us to capture the potential nonlinear relationships between globulin, AGR and depression, providing a more nuanced understanding than using a linear model alone. Additionally, we conducted subgroup analyses to distinguish the associations in various subgroups of cancer and non-cancer populations, revealing nuanced insights into how these biomarkers interact with depression across different lifestyles and physiological conditions. Adjusting for potential confounders, including demographic, lifestyle, and health-related variables, strengthened the validity of our findings. These methodological strengths underscore the robustness of our conclusions and emphasize the importance of considering globulin levels and AGR in future depression research, particularly among cancer patients.

Despite uncovering valuable findings, this study has several limitations that should be considered. First, due to the cross-sectional design, we cannot infer a causal relationship between globulin levels, AGR, and depression. Although our findings highlight strong associations that align with known pathways linking inflammation and depression. For example, elevated globulin levels reflect chronic immune activation, which may compromise blood-brain barrier integrity and promote neuroinflammation via cytokines (52). In contrast, a low AGR (driven by hypoalbuminemia) indicates malnutrition or metabolic dysfunction, which may exacerbate depressive symptoms through oxidative stress or hypothalamic-pituitary-adrenal (HPA) axis dysregulation. However, causal relationships should be interpreted with caution, as depression itself may alter inflammatory profiles and nutritional status, making reverse causality a possibility. Therefore, prospective longitudinal studies are needed in the future to establish temporal relationships and determine causality. Secondly, due to database limitations, the identification of cancer and depression was based on self-reported questionnaires. Although the PHQ-9 has high sensitivity and specificity (53) and its validity and performance have been confirmed in cancer patients (54), the overlap of somatic symptoms may still introduce bias. Future studies should incorporate multiple assessment methods and integrate medical records to accurately ascertain the occurrence of depression and cancer, thereby improving research accuracy. Furthermore, although we adjusted for demographic factors, lifestyle variables, and common comorbidities, we acknowledge that due to limitations in available data, some confounding factors that are difficult to identify (such as medication use, past psychiatric history, and cancer severity) may influence the observed associations. Future studies incorporating these variables will be necessary to refine the model. Third, due to differences in populations and healthcare systems, the generalizability of our findings to populations outside the United States may be limited.

## 5 Conclusions

In conclusion, our study reveals significant associations of globulin and AGR with depression, showing distinct patterns in cancer and non-cancer populations. These findings underscore the necessity for further research to explore the underlying mechanisms behind the differences in the association between serum proteins and mental health outcomes in different populations, as well as the potential role of these differences in the development of depression. Additionally, monitoring globulin levels and AGR may provide valuable insights for the early detection and management of depression in clinical settings, particularly in more vulnerable cancer populations.

## Data Availability

Publicly available datasets were analyzed in this study. This data can be found here: https://www.cdc.gov/nchs/nhanes/index.htm.
